# Cellular Therapy for In Utero Repair of Myelomeningocele (the CuRe trial): neurosurgical repair video

**DOI:** 10.3171/2026.1.FOCVID25229

**Published:** 2026-04-01

**Authors:** Elizabeth Reynolds, Payam Saadai, Cameron Sadegh, Amelia S. McLennan, Erin G. Brown, Jamie E. Anderson, Priyadarsini Kumar, Aijun Wang, Diana L. Farmer, Marike Zwienenberg

**Affiliations:** 1 Department of Surgery,; 2 Department of Neurological Surgery,; 3 Department of Obstetrics and Gynecology, and; 4 Center for Bioengineering in Medicine, University of California, Davis Health, Sacramento, California

**Keywords:** spina bifida, myelomeningocele, fetal, stem cells

## Abstract

The Management of Myelomeningocele Study (MOMS) established that prenatal repair of myelomeningocele (MMC) improved the rate of ventriculoperitoneal shunt insertion for the treatment of hydrocephalus, compared to postnatal repair. While there was mild improvement of motor function, most children were unable to ambulate independently. Therefore, the ongoing phase 1/2a clinical trial Cellular Therapy for In Utero Repair of Myelomeningocele (CuRe trial) aims to further improve distal neurological function using placenta-derived mesenchymal stem/stromal cells seeded on an extracellular matrix (PMSC-ECM) to augment open fetal repair of MMC. Here, the authors present a video of the neurosurgical repair using the PMSC-ECM.

The video can be found here: https://stream.cadmore.media/r10.3171/2026.1.FOCVID25229

## Transcript

We present the case of an otherwise healthy 28-year-old G2P1 female carrying a fetus with myelomeningocele at L5 and an associated Chiari II malformation. She was enrolled in the CuRe trial [NCT04652908] according to MOMS criteria.^1–3^ Open fetal surgery with the placement of placenta-derived mesenchymal stem/stromal cells seeded on an extracellular matrix, referred to as the PMSC-ECM,^4–6^ occurred at 24 weeks and 3 days’ gestational age.

Shown here are representative images of the defect on prenatal ultrasound and MRI, which was estimated to span from L5 to S4. On the screen to the right, you can see the hindbrain herniation associated with a Chiari II malformation. The ventricles measured 10.5 and 8 mm on the right and left, respectively.

Prenatal ultrasound demonstrated movement of the bilateral lower extremities.

### 1:12 Patient Positioning.

An epidural and Foley catheter are placed. The patient is positioned in lithotomy. Our multidisciplinary team is shown here.

### 1:22 Surgical Procedure.

A low transverse incision is made through which the uterus is exposed. Placenta and fetal location are once again assessed using ultrasound. Fetal heart rate and temperature are monitored at least every 5 minutes until the maternal skin is closed. This is performed by a maternal-fetal medicine physician and sonographer.

The myelomeningocele defect is exposed through a (3-inch) hysterotomy.

### 1:56 Neurosurgical Repair.

The neural placode is dissected circumferentially and disconnected from the skin. Retention sutures are temporarily placed in the fetal skin for retraction and improved visualization of the neural placode and dural junction zone.

A small dural opening is made using microscissors. Using an angiocatheter, a small amount of saline is then injected below the dura to perform hydrodissection and aid in mobilization of the dura. Subsequently, the dura is incised circumferentially and elevated off the underlying fascia. The epidural space is exposed.

### 3:14 Preparation for and Placement of the PMSC-ECM.

The dura is reapproximated cranially and caudally to create a pocket for placement of the PMSC-ECM.

The PMSC-ECM is then topically applied onto the placode, with stem cells in direct contact with the spinal cord tissue and tailored to the defect. Excess is trimmed. The dura is reapproximated over the PMSC-ECM.

If a primary closure of the dura over the PMSC-ECM is not feasible, the PMSC-ECM is used as an inlay duraplasty. If dural closure is deemed inadequate, then dural closure is supplemented by a collagen graft as an onlay duraplasty. Myofascial flaps may be raised to further support the closure at the discretion of the neurosurgeons.

When possible, a primary closure of the dura over the PMSC-ECM is performed. A separate representative video of such a case is shown here.

The fetal skin is closed primarily. Finally, the maternal hysterotomy and abdomen are closed.

### 7:01 Postoperative Course.

The patient recovered well postoperatively. The neonate was delivered by C-section at 34 weeks and 3 days.

Postoperative and neonatal images demonstrate reversal of hindbrain herniation and decreased size of the ventricles. The repaired site appeared to be covered with a robust layer of subcutaneous tissue, while the CSF space around the neural placode appeared preserved (red arrow).

This is an ongoing phase 1/2a clinical trial assessing the safety and preliminary efficacy of the experimental PMSC-ECM product applied at the time of open fetal MMC repair. Motor outcomes will be formally evaluated at 30 months of age.

## Acknowledgments

We acknowledge many additional individuals who contribute to this ongoing clinical trial, including members of the University of California, Davis Fetal Care and Treatment Center; the Center for Bioengineering in Medicine; the University of California, Davis Good Manufacturing Practice Facility and product manufacturing team; the University of California, Davis Clinical and Translational Science Center; Shriners Children’s Hospital; and the California Institute for Regenerative Medicine.

Funding was received from the California Institute for Regenerative Medicine (CIRM) (CLIN2-12129 to A.W. and D.L.F.) and Shriners Hospitals for Children (72008-NCA-21 and 70012-NCA-23 to A.W. and D.L.F.).

## Disclosures

A. Wang reported a patent 10,058,572 issued, a patent 11,583,557 issued, and a patent for 18/098,631 pending. D. Farmer reported a patent for UC Davis pending.

## Author Contributions

Primary surgeon: Saadai, Zwienenberg. Assistant surgeon: Sadegh, McLennan, Anderson, Farmer. Editing and drafting the video and abstract: Reynolds, Saadai, Sadegh, Farmer, Zwienenberg. Critically revising the work: Reynolds, Saadai, Sadegh, Brown, Farmer, Zwienenberg. Reviewed submitted version of the work: Reynolds, Saadai, Sadegh, Brown, Anderson, Wang, Farmer, Zwienenberg. Approved the final version of the work on behalf of all authors: Reynolds, Zwienenberg. Supervision: Saadai, Wang. Lead product manufacturing: Kumar.

## Supplemental Information

### Patient Informed Consent

The necessary patient informed consent was obtained in this study.
